# Influence of stripe rust infection on the photosynthetic characteristics and antioxidant system of susceptible and resistant wheat cultivars at the adult plant stage

**DOI:** 10.3389/fpls.2015.00779

**Published:** 2015-09-28

**Authors:** Yang-Er Chen, Jun-Mei Cui, Yan-Qiu Su, Shu Yuan, Ming Yuan, Huai-Yu Zhang

**Affiliations:** ^1^College of Life Sciences, Sichuan Agricultural UniversityYa’an, China; ^2^Tongwei Group Co. LtdChengdu, China; ^3^College of Resources, Sichuan Agricultural UniversityChengdu, China

**Keywords:** stripe rust, antioxidant enzyme, chlorophyll fluorescence, photosystem II, *Triticum aestivum* L.

## Abstract

Wheat stripe rust (*Puccinia striiformis* f. sp. *tritici, Pst*), is one of the most serious diseases of wheat (*Triticum aestivum* L.) worldwide. To gain a better understanding of the protective mechanism against stripe rust at the adult plant stage, the differences in photosystem II and antioxidant enzymatic systems between susceptible and resistant wheat in response to stripe rust disease (*P. striiformis*) were investigated. We found that chlorophyll fluorescence and the activities of the antioxidant enzymes were higher in resistant wheat than in susceptible wheat after stripe rust infection. Compared with the susceptible wheat, the resistant wheat accumulated a higher level of D1 protein and a lower level of reactive oxygen species after infection. Furthermore, our results demonstrate that D1 and light-harvesting complex II (LHCII) phosphorylation are involved in the resistance to stripe rust in wheat. The CP29 protein was phosphorylated under stripe rust infection, like its phosphorylation in other monocots under environmental stresses. More extensive damages occur on the thylakoid membranes in the susceptible wheat compared with the resistant wheat. The findings provide evidence that thylakoid protein phosphorylation and antioxidant enzyme systems play important roles in plant responses and defense to biotic stress.

## Introduction

Common wheat (*Triticum aesitivum* L.) is the major cereal crop in the world, and its yield and grain quality are highly impacted by various fungal diseases such as *Fusarium* head blight, powdery mildew (*Blumeria graminis*), stem rust (*Puccinia graminis*), and stripe rust (*Puccinia Striiformis*; Pei et al., 2015). Among the three main rusts, wheat stripe rust, is one of the most serious wheat diseases worldwide, which is the most destructive disease in the northwestern and southwestern wheat-growing regions in China (Wan et al., 2004; Wellings, 2011; Ma et al., 2013). Wheat production can be greatly reduced or even completely destroyed when seedlings are infected and the disease continues to spread during the growing season (Chen, 2005). Growing resistant cultivars is the most effective, safest, economical, and environmentally sound approach to control the wheat stripe rust (Chen, 2005; Dodds and Rathjen, 2010). However, cultivars with race-specific resistance usually become susceptible within a few years due to the rapid evolution of virulent races of Pst (Line and Chen, 1995). Therefore, it is essential to create strategies for improving disease resistance in wheat.

Currently, resistance to stripe rust in wheat has been broadly categorized into the all-stage resistance (also called seedling resistance, detected at the seedling stage) and the adult plant resistance (APR, detected only at the adult plant stage; Chen, 2005). Biotic stress usually stimulates the production of reactive oxygen species (ROS), such as the superoxide anion ($O_{2}^{\cdot –}$), hydrogen peroxide (H_2_O_2_) and hydroxyl radicals (OH⋅Suzuki and Mittler, 2006). The production of ROS is one of the earliest responses of plant tissues to elicitors and attack by pathogens. Previous studies on histochemical methods have indicated that wheat stripe rust can induce the generation of H_2_O_2_ and $O_{2}^{\cdot –}$ at the seedling or adult plant stage (Wang et al., 2007; Zhang et al., 2012). However, excessive levels of ROS potentially damage the plant cell unless they are detoxified by the antioxidative enzymes (Agarwal et al., 2005). These studies on wheat response to stripe rust indicated that the antioxidant enzymes play an important role in resistance to biotic stress (Asthir et al., 2010; Anahid et al., 2013). Although ROS are associated with effective plant resistance responses (especially in incompatible biotrophic pathogen–plant interactions), some necrotrophic pathogens may generate ROS to induce cell death, which facilitates subsequent spread of the pathogen (Tiedemann, 1997). At present, there are few studies on comparing of ROS and antioxidative enzymes in susceptible and resistant wheat cultivars upon the inoculation with stripe rust.

It is well known that more than 90% of crop biomass is derived from photosynthetic products. Although a lack of correlation between photosynthesis and plant yield has been reported frequently, some study suggests that enhanced photosynthesis may increase the yield when other genetic factors remain unchanged (Evans and Dunstone, 1970; Long et al., 2006; Makino, 2011). Photosynthetic activities are affected by many abiotic and biotic stresses. However, so far, most studies on wheat photosynthesis under environment stresses focused on abiotic stresses (Lu and Zhang, 1998; Shah and Paulsen, 2003; Yang et al., 2008). In contrast, only a few studies have been conducted on the relationship between the photosynthesis and biotic stresses. Even though a previous study indicated that pathogen infection may result in changes in photosynthesis (Wang et al., 2000), the detailed effects of wheat stripe rust on photosystem II (PSII) are poorly understood.

In order to reveal the relationship between the PSII and the resistance levels of wheat to stripe rust, we compared changes in the ROS contents, the activities of antioxidant enzymes, chlorophyll fluorescence, thylakoid membrane protein levels, and protein phosphorylation in susceptible and resistant wheat cultivars. The present results are expected to provide a better understanding of wheat resistance mechanisms against stripe rust infections, and thus to improve the yield of wheat.

## Materials and Methods

### Plants, Pathogens, and Inoculation

Two wheat (*Triticum aestivum* L.) cultivars, namely Sy95-71 and CN19, and the Chinese *Pst* race CY32 were used in the present study. The wheat cv. Sy95-71 and CN19 are susceptible (IT = 4, high susceptibility) and resistant (IT = 0; nearly immune) to race CY32 at the adult plant stage (Luo et al., 2005).

For the adult plant experiments, five vernalized seedlings were grown in a 20-cm diameter pot filled with pre-fertilized soil. The plants were grown to the boot stage in the greenhouse under 16 h light (sodium light, 240 μmol of photons m^-2^ s^-1^) and 8 h of darkness, and the greenhouse temperature were maintained at 22°C with light and 12°C in darkness. Inoculations were performed by applying fresh urediniospores to the flag leaves of adult wheat plants with a fine paintbrush until the whole leaf surface was wet without run-off. Parallel control inoculations (CK) were treated in the same way with tap water. Subsequently, inoculated plants were maintained at high humidity at 10°C for 24 h in the darkness and then returned to the greenhouse. Inoculated and control leaves were harvested at 72 h post-inoculation (hpi) for various analyses. The remaining plants were continually grown to assess the stripe rust phenotypes. Three independent biological replications were performed for each treatment.

### Chlorophyll Contents, Photosynthetic Rate, Leaf Water Status, and Total Protein Content

Chlorophyll *a* and *b* contents were assayed according to the method of Porra et al. (1989). Fresh leaves (0.5 g) were cut, homogenized and extracted with 80% acetone at room temperature. The extracts were filtered through two layers of filter paper. After filtering, the absorbance of the solution was read at 645 and 663 nm using a spectrophotometer (Hitachi-U2000, Tokyo, Japan). The net photosynthetic rate (*P*_n_) was determined using an open gas analysis system as previously described (Liu et al., 2009). The relative water content (RWC) of the leaf was calculated using the following formula (Li et al., 2014): RWC = (fresh weight - dry weight)/(turgid weight – dry weight) × 100%. The turgid weight was determined after placing the leaves in distilled water under dark conditions at 4°C overnight, until they reached a constant weight. The dry weight was obtained 24 h after placing the turgid leaves in an oven at 85°C. The total soluble protein content was measured as described previously (Lowry et al., 1951). Fresh leaves (0.5 g) were homogenized with 5 ml sodium phosphate buffer (pH 7.2) and then centrifuged for 10 min at 4°C. Supernatants were used for the analysis of soluble protein using a UV spectrophotometer.

### Determinations of Lipid Peroxidation and Electrolyte Leakage

The degree of lipid peroxidation was estimated based on the malondialdehyde (MDA) contents as previously described with minor modification (Luo et al., 2009). Fresh leaf tissues (0.5 g) were homogenized in 5 mL 5% (w/v) tri-chloro acetic acid (TCA). The homogenate was centrifuged at 4°C for 10 min at 5,000 *g*. A volume of 2 mL of the supernatant was combined with 2 mL of 5% TCA containing 0.67% thiobarbituric acid (TBA). The assay mixture was incubated at 95°C for 30 min and then rapidly cooled on ice. The mixture was centrifuged at 5,000 *g* for 10 min at 4°C. The absorbance of the supernatant was monitored at 532 nm and corrected for non-specific turbidity by subtracting the absorbance at 600 nm. Electrolyte leakage was measured according to the method of Chen et al. (2015). After measuring the conductivity, the samples were heated in water bath at 95°C for 15 min to achieve 100% electrolyte leakage.

### Assay of ROS

Visual detection of the superoxide anion radicals ($O_{2}^{\cdot –}$) and H_2_O_2_ levels was performed using nitro blue tetrazolium (NBT) and 3,3-diaminobenzidine (DAB), respectively, as described previously with some modifications (Yang et al., 2004). The inoculated and control leaves were excised at the base with a razor blade and immersed in a solution containing 6 mM NBT, 50 mM Hepes buffer (pH 7.5) for 2 h or 5 mM DAB dissolved in 10 mM 4-Morpholineethanesulfonic acid (MES) (pH 3.8) for 8 h in the darkness. Detached leaves were then fixed and decolorized in boiling ethanol (90%) for 0.5–2 h. At least three leaves were used for each treatment.

The H_2_O_2_ content was measured as described previously (Okuda et al., 1991). Approximately 0.5 g of fresh leaf tissue was cut into small pieces and ground in an ice bath with 5 mL 0.1% (w/v) TCA. After centrifugation (20 min, 12,000 *g*), 0.5 mL of supernatant was added to 0.5 mL 10 mM potassium phosphate buffer (pH 7.0) and 1 mL of 1 M potassium iodide. The absorbance of the supernatant was recorded at 390 nm. Finally, the content of H_2_O_2_ was calculated using a standard curve plotted with known concentrations of H_2_O_2_. The $O_{2}^{\cdot –}$ content was determined as described previously by monitoring the nitrate formation from hydroxyl amine (Elstner and Heupel, 1976).

### Determination of Antioxidative Enzyme Activities

The enzymes were extracted at 4°C from 0.5 g of fresh leaf tissues using a chilled mortar and pestle with 5 mL ice-cold 25 mM Hepes buffer (pH 7.8) containing 0.2 mM ethylenediaminetetraacetic acid, 2 mM ascorbate and 2% polyvinylpyrrolidone (PVP). The extracts were centrifuged at 12,000 *g* for 30 min at 4°C. After centrifugation, the supernatant was used for the enzyme assays. Peroxidase (POD), superoxide dismutase (SOD), catalase (CAT), ascorbate peroxidase (APX), glutathione peroxidase (GPX), and glutathione reductase (GR) were assayed as previously described (Chen et al., 2015).

### Chlorophyll Fluorescence Visualization

Chlorophyll fluorescence images were recorded at room temperature using a modulated imaging fluorometer (the Imaging PAM M-Series Chlorophyll Fluorescence System, Heinz-Walz Instruments, Effeltrich, Germany) according to the instructions provided by the manufacturer. Infected and control wheat samples were dark adapted for 30 min prior to the fluorescence measurements. Values of *F*_0_ (minimum fluorescence yield) and *F*_m_ (maximum fluorescence yield) were averaged to improve the signal-to-noise ratio. The image data acquired in each experiment were normalized to a false color scale. The maximum efficiency of PSII photochemistry in the dark-adapted state (*F*_v_/*F*_m_), the photochemical quenching (qP), the quantum yield of PSII electron transport (ΦPSII), and the non-photochemical quenching coefficient (NPQ) were visualized according to the method of Maxwell and Johnson (2000).

### Protein Gel Blotting Analysis

The isolation of thylakoid membrane from wheat was performed as described (Chen et al., 2009). Thylakoid membrane proteins were separated by SDS-PAGE (6% acrylamide stacking gel + 15% separation gel + 6 M urea). Then immunodetection was performed on thylakoid membranes according to the method as described previously (Chen et al., 2009). For the anti-phosphothreonine antibody, purchased from New England Biolabs (Cell Signaling, Ipswich, MA, USA), and the membrane was blocked with 5% BSA (Sigma Chemical Co. St. Louis, MO, USA). Antibodies against Lhca1, Lhca2, Lhca3, Lhca4, D1, D2, CP43, Lhcb1, Lhcb2, Lhcb3, Lhcb4, Lhcb5, Lhcb6, and Rubisco were obtained from Agrisera (Umea, Sweden). The membranes were then incubated with horseradish peroxidase-conjugated secondary antibody (Bio-Rad Comp. Hercules, CA, USA) and developed using a chemiluminescent detection system (ECL, GE Healthcare). Quantification of the immunoblots was done using Quantity One software (Bio-Rad Comp. Hercules, CA, USA).

### Transmission Electron Microscopy

Pieces of leaf tissue from control and infected plants were fixed immediately with 3% glutaraldehyde in 0.1 sodium cacodylate buffer (pH 6.9) at 4°C overnight, post-fixed with 1% osmium tetroxide, dehydrated in series acetone and embedded in Epon 812, as described previously (Liu et al., 2009). Thin sections cut with an ultramicrotome (Ultracut F-701704, Reichert-Jung, Austria) were stained with uranyl acetate and observed in a Transmission Electron Microscope (TEM H600, Hitachi) operating at 100 kV.

### Statistical Analysis

SPSS 19.0 (IBM, Chicago, IL, USA) statistical software was used for the statistical evaluation of the data. All results were presented with mean ± SD from three independent biological replicates. The means were compared using Duncan’s multiplication range test. Differences between all four types of treatments were considered to be statistically significant when *P* < 0.05.

## Results

### Symptoms after Inoculation

**FIGURE 1:**
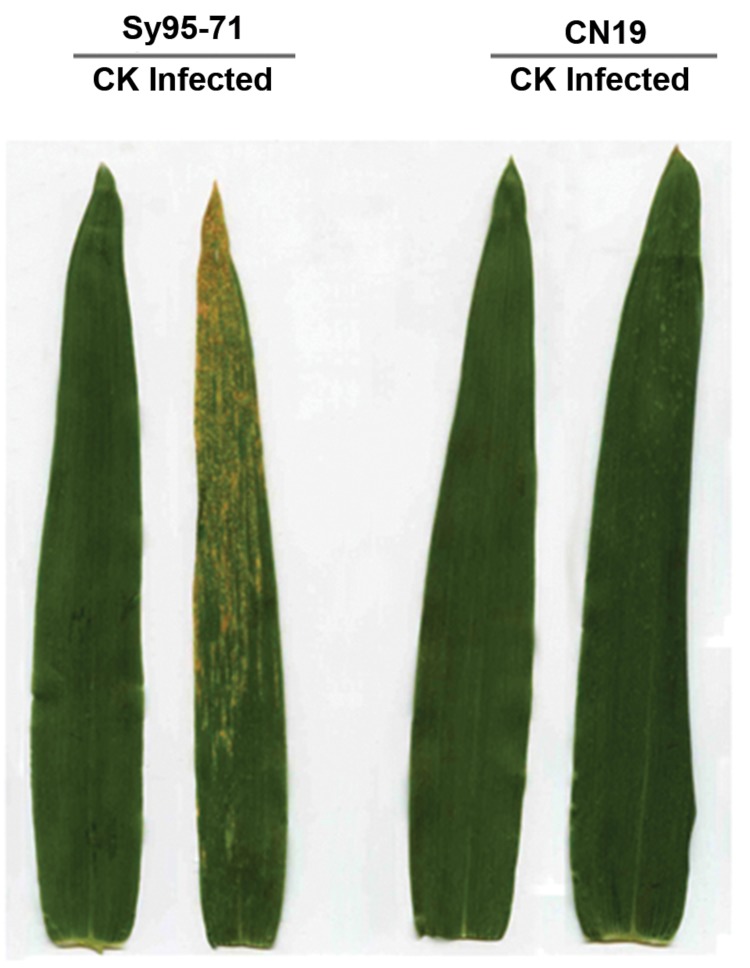
**Stripe rust disease symptoms at 14 days post inoculation (dpi) on the susceptible (Sy95-71) and resistant (CN19) wheat cultivars at the adult plant stage.** CK, un-inoculated leaves.

Adult plants of Sy95-71 and CN19 showed different symptoms at 14 days post-inoculation (dpi) with race CY32 (**Figure 1**). Stripe rust uredia were visible on the flag leaves in the susceptible wheat Sy95-71, representing a disease severity of 95% compared with the un-inoculated control of Sy95-71. However, no sporulation appeared on inoculated leaves in the resistant wheat cultivar CN19. Therefore, Sy95-71 was susceptible, while CN19 was highly resistant against to the stripe rust at the adult plant stage.

### Effect of Wheat Stripe Rust on the Chlorophyll (Chl) Content, RWC, and Total Protein Content

**FIGURE 2:**
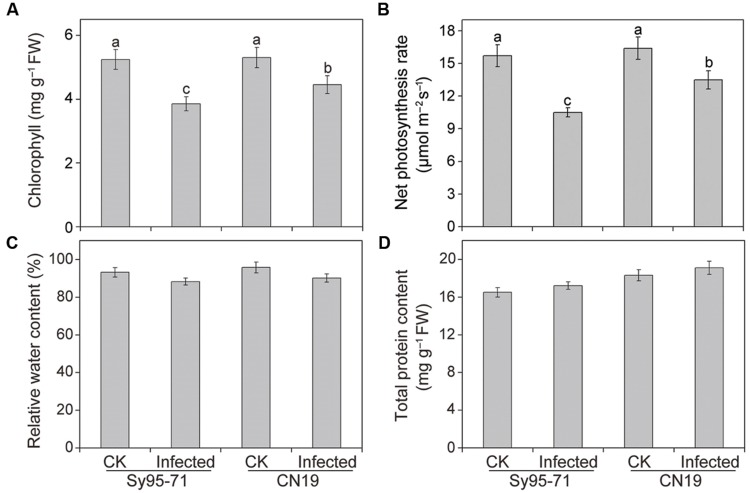
**Chlorophyll **(A)**, net photosynthetic rate **(B)**, relative water content (RWC) **(C)**, and total protein content **(D)** in inoculated and un-inoculated leaves of Sy95-71 and CN19.** Error bars represent the standard deviation based on three biological replicates. Different letters depict significant differences between the susceptible and resistant wheat cultivars (*P* < 0.05). Statistical analysis was performed using one-way ANOVA followed by Duncan’s multiple range test. CK, un-inoculated wheat plants.

There was no significant difference in the Chl, *P*n, RWC, and total protein content between the control plants of the susceptible and resistant wheat (**Figure 2**). In CN19, the wheat stripe rust fungal infection caused a decrease in the Chl content of 17% compared with the CN19 control (**Figure 2A**). However, a marked decrease in Chl content was observed in the susceptible wheat Sy95-71 after inoculation at the boot stage, reaching 27% when compared with the Sy95-71 control. The infected Sy95-71 also had a more significant decrease in the photosynthesis rate compared with the infected CN19 at 72 hpi. Apparently, the *Pst* infection can regulate photosynthetic rates of infected leaves in both Sy95-71 and CN19 (**Figure 2B**). However, after inoculation, the RWC and total protein content were not significantly different between the susceptible and the resistant wheat (**Figures 2C,D**).

### *Pst* Infection Induces ROS Accumulation and Lipid Peroxidation in Leaves

**FIGURE 3:**
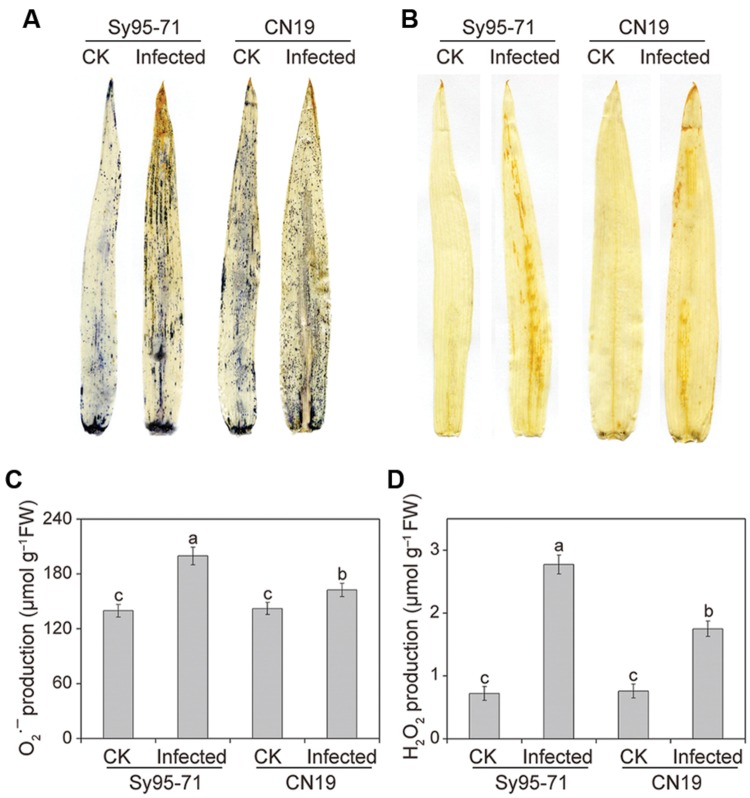
**Measurement of reactive oxygen species (ROS) after stripe rust infection.** Histochemical assays for superoxide anion radicals ($O_{2}^{\cdot –}$) and hydrogen peroxide (H_2_O_2_) by nitro blue tetrazolium (NBT) **(A)** and 3,3-diaminobenzidine (DAB) **(B)** staining, respectively. Then, $O_{2}^{\cdot –}$ production **(C)** and the H_2_O_2_ content **(D)** were measured. Values are means ± SD from three independent biological replicates. Different letters depict significant differences between the susceptible (Sy95-71) and resistant (CN19) wheat cultivars (*P* < 0.05). CK, un-inoculated wheat plants.

The induction of $O_{2}^{\cdot –}$ and H_2_O_2_ by *Pst* inoculation in the CN19 and Sy95-71 plants at the boot stage was analyzed by histochemical staining with NBT and DAB, respectively. No significant difference was detected among the controls of CN19 and Sy95-71 (**Figures 3A,B**). Interestingly, both $O_{2}^{\cdot –}$ and H_2_O_2_ were upregulated in infected leaves in both susceptible and resistant wheat, compared with the control plants. In comparison, the accumulation of $O_{2}^{\cdot –}$ and H_2_O_2_ was more pronounced in the infected Sy95-71 than that of the infected CN19.

**FIGURE 4:**
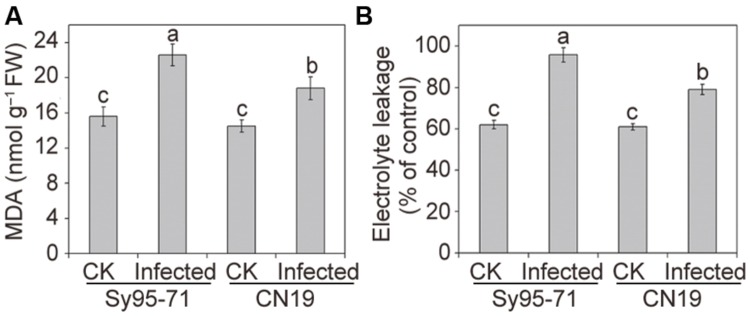
**Effects of stripe rust infection on the malondialdehyde (MDA) content **(A)** and electrolyte leakage **(B)** of the susceptible (Sy95-71) and resistant (CN19) wheat cultivars.** Bars represent standard deviations of three independent biological replicates and values followed by different letters are significantly different at *P* < 0.05 according to Duncan’s multiple range test. CK, un-inoculated wheat plants.

Quantitative levels of H_2_O_2_ and $O_{2}^{\cdot –}$ produced in the inoculated and un-inoculated leaves were determined further. After infection, the level of $O_{2}^{\cdot –}$ accumulation in CN19 was slightly higher than that observed in the control plants (**Figure 3C**). In Sy95-71, stripe rust fungal inoculation resulted in a significant increase in the $O_{2}^{\cdot –}$ content compared with that of the controls and CN19 (*P* < 0.05). Similar results were observed for the level of H_2_O_2_. $O_{2}^{\cdot –}$ and H_2_O_2_ levels in Sy95-71 increased by 43 and 284%, respectively, after inoculation compared with the control (**Figures 3C,D**). In addition, we examined the degree of oxidative damage in leaves of wheat subjected to *Pst* infection by determining the amount of lipid peroxidation (Barclay and Mckersie, 1994). As shown in **Figure 4A**, the stripe rust fungal infection raised the concentration of MDA about 29 and 47% in CN19 and Sy95-71, respectively, when compared with the respective controls. Similarly, the electrolyte leakage in inoculated Sy95-71 was also higher than that of inoculated CN19 (**Figure 4B**).

### Effect of Stripe Rust on the Activities of Antioxidant Enzymes

**FIGURE 5:**
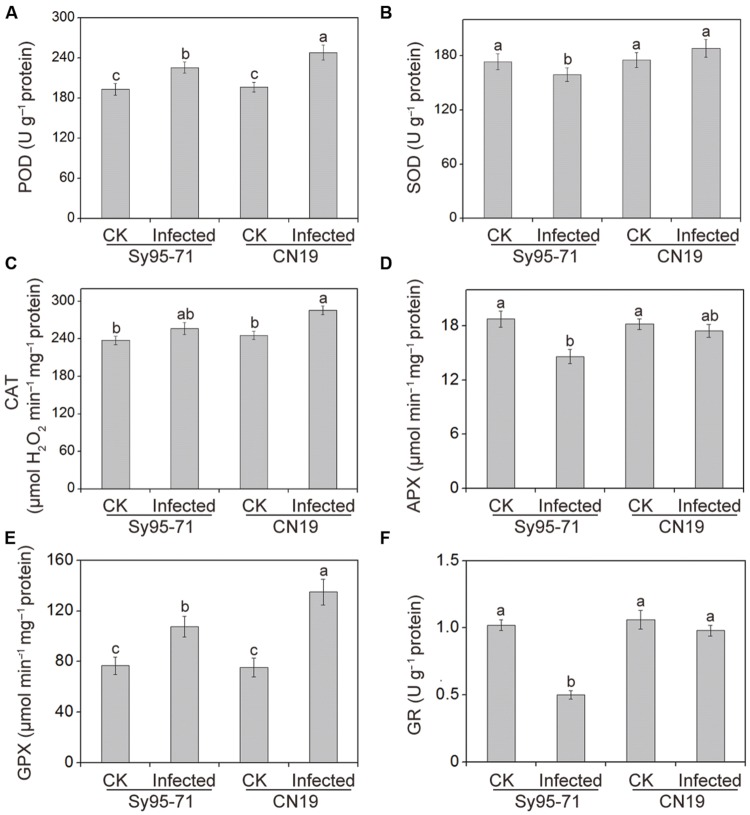
**Effects of stripe rust infection on the POD, Peroxidase **(A)**; SOD, superoxide dismutase **(B)**; catalase CAT, catalase; **(C)**; APX, ascorbate peroxidase **(D)**; GPX, glutathione peroxidase **(E)**; and GR, glutathione reductase **(F)** in the susceptible (Sy95-71) and resistant (CN19) wheat cultivars.** Bars represent standard deviations of three independent biological replicates and values followed by different letters are significantly different at *P* < 0.05 according to Duncan’s multiple range test. CK, un-inoculated wheat plants.

The effects of *Pst* infection on the activities of antioxidant enzymes in the susceptible and resistant wheat are presented in **Figure 5**. The antioxidant enzyme activities in the un-inoculated plants of Sy95-71 and CN19 showed no significant difference (**Figure 5**). After inoculation, the POD, CAT, and GPX activities increased in Sy95-71 and CN19 compared with their respective controls. However, a more obvious increase was observed in CN19, especially with respect to GPX activity. In Sy95-71 and CN19, infections caused significant increases in GPX activities of 41 and 79%, respectively (**Figure 5E**). However, after infection, SOD activity decreased slightly in Sy95-71 but was not significantly changed in CN19 compared with the respective controls (**Figure 5B**). In addition, we found that APX and GR activities decreased in Sy95-71 and CN19 infected with *Pst* (**Figures 5D,F**). A more pronounced decrease was observed in Sy95-71 after infection. There was a 50% decrease in GR activity in Sy95-71 exposed to the stripe rust (**Figure 5F**).

### Effect of Wheat Stripe Rust Inoculation on Chlorophyll Fluorescence

**FIGURE 6:**
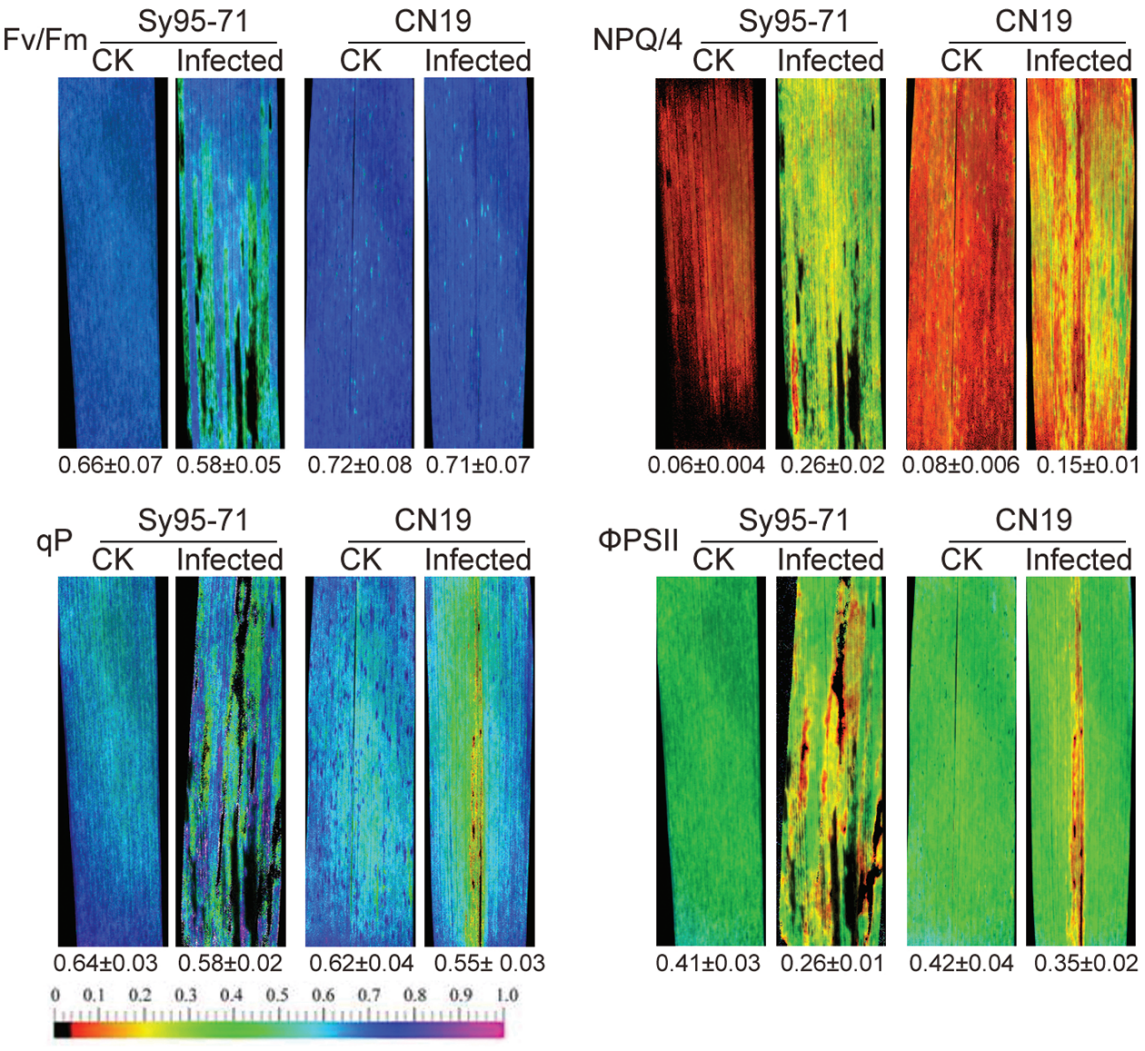
**Effects of stripe rust infection on chlorophyll fluorescence parameters (*F*_v_/*F*_m_; qP, photochemical quenching; NPQ/4, non-photochemical quenching coefficient; and ΦPSII, quantum yield of PSII electron transport) in Sy95-71 and CN19.** Quantitative values (±SD) are shown below the individual fluorescence images. CK, un-inoculated wheat plants.

The chlorophyll fluorescence of wheat leaves inoculated with *Pst* was examined by a modulated imaging fluorometer. In CN19, the maximum photochemical efficiency of PSII in the dark-adapted state (*F*_v_/*F*_m_) had no significant decrease in the inoculated leaves compared with the un-inoculated leaves (**Figure 6**). Compared with CN19, Sy95-71 exhibited a significant decrease in the *F*_v_/*F*_m_ value after the inoculation. Furthermore, we found that the NPQ, the quantum yield of ΦPSII, and the qP values were also influenced significantly in inoculated leaves of Sy95-71 compared with the un-inoculated leaves (**Figure 6**). These obvious effects were not observed in the infected leaves of CN19.

### Effect of Stripe Rust on the Thylakoid Protein Content and Phosphorylation

**FIGURE 7:**
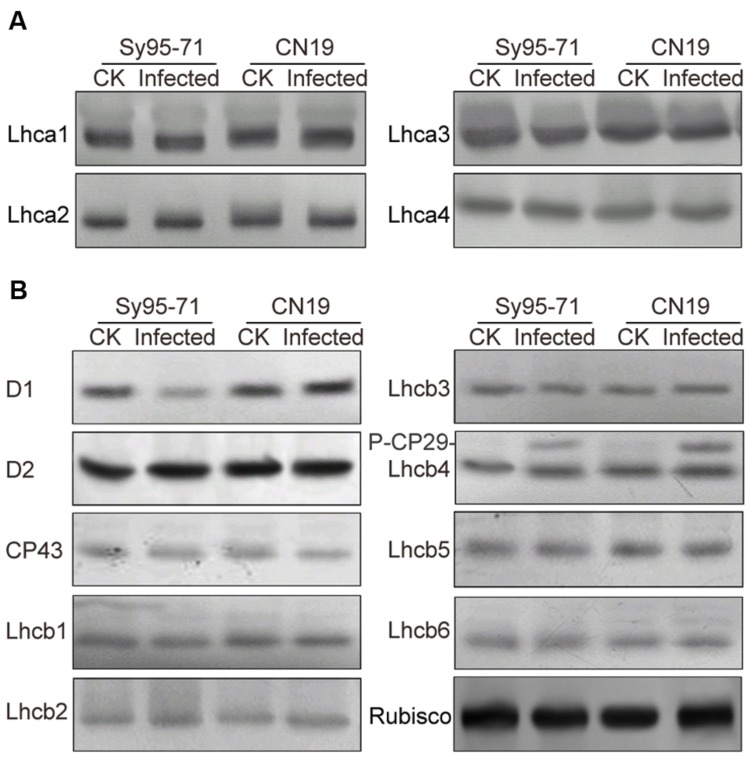
**Immunoblot analyses of thylakoid proteins in inoculated and un-inoculated wheat plants (Sy95-71 and CN19).** Immunoblot analyses of thylakoid membrane proteins were performed using antibodies specific for representative PSI, photosystem I **(A)**; PSII, photosystem II **(B)**; and Rubisco proteins. One microgram of total chlorophyll was loaded into each electrophoretic lane. CK, un-inoculated wheat plants. Rubisco was used as the standard reference for western blotting.

**FIGURE 8:**
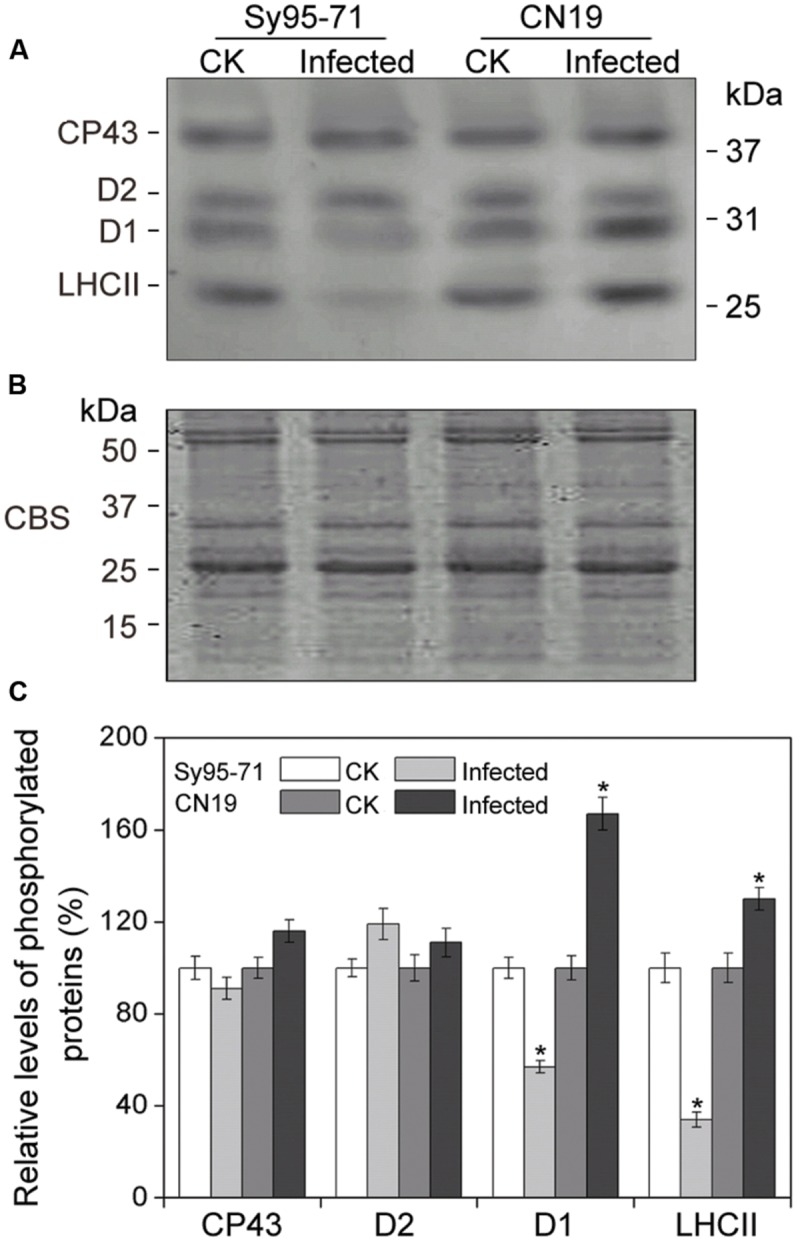
**Thylakoid protein phosphorylation after wheat stripe rust infection of the susceptible (Sy95-71) and resistant (CN19) wheat cultivars.** Thylakoid proteins extracted from the inoculated and un-inoculated wheat plants were fractionated by SDS-PAGE in 12% acrylamide separation gel with 6 M urea. Immunoblot analysis of thylakoid membrane proteins was performed using anti-phosphothreonine antibodies **(A)**. Loading was based on an equal amount of chlorophyll (1 μg chlorophyll). The SDS-PAGE results after Coomassie blue staining (CBS) are shown in the bottom panel **(B)**. CK, un-inoculated wheat plants. **(C)** Quantification of immunoblot data. Results are presented relative to the amount of respective CK (100%). Asterisks indicates statistically significant differences at the *P* < 0.05 level. Values are means ± SD from three independent biological replicates.

To determine whether a reduction in the abundance of PSII subunits occurred in the susceptible wheat, the thylakoid polypeptide composition was investigated by the immuno-blotting (**Figure 7**). The levels of four major PSI proteins showed no detectable changes after the infection (**Figure 7A**). Although there was no obvious difference in the level of almost all PSII proteins between inoculated and un-inoculated wheat, changes in the D1 and Lhcb4 (CP29) proteins were observed in Sy95-71 and CN19 after the infection (**Figure 7B**). Compared with the control, infection resulted in a decrease in the D1 protein in Sy95-71. By contrast, the level of D1 was increased in CN19 after the infection compared with the control. We also found that the level of the D2 protein showed no obvious difference between Sy95-71 and CN19 (**Figure 7A**). Interestingly, although the level of the CP29 protein did not change in any of the plants, CP29 protein was phosphorylated in inoculated wheat plants, especially in CN19 (**Figure 7B**). Further analyses of thylakoid membrane protein phosphorylation were performed with the anti-phosphothreonine antibody. As shown in **Figure 8A**, thylakoid protein phosphorylation did not show obvious differences after the infection, although phosphorylated-D1 (P-D1) and P-LHCII presented some differences. In Sy95-71, the infection resulted in a decrease in the level of P-D1 and P-LHCII compared with the control plants. In contrast, more levels of phosphorylated D1 and light-harvesting complex II (LHCII) were found in the infected CN19 than that of the non-inoculated control (**Figure 8**).

### Alterations in the Thylakoid Ultrastructure of Plants Infected with Stripe Rust

**FIGURE 9:**
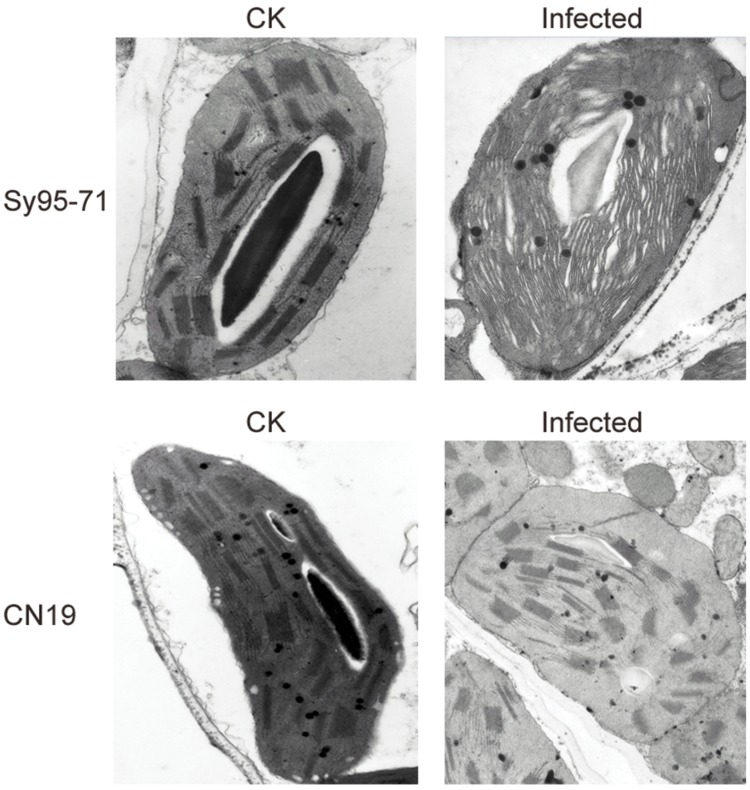
**Transmission electron microscope analysis of chloroplasts after stripe rust infection of the susceptible (Sy95-71) and resistant (CN19) wheat cultivars.** CK, un-inoculated wheat plants.

To further investigate effects of the stripe rust infection on PSII structures, the thylakoid membrane ultrastructure was analyzed with a transmission electron microscopy. The infection in Sy95-71 resulted in a significant reduction in the stacking of the grana, and the thylakoid structure had become fibrous compared with the control. However, the infection of CN19 did not induce any obvious changes in the thylakoid membrane structure compared with the control (**Figure 9**).

## Discussion

It is well known that wheat stripe rust is one of the most destructive diseases of wheat, which can cause severe yield losses when susceptible cultivars are grown in the field (Wan et al., 2004; Chen, 2005). The use of resistant cultivars is the most cost-effective and environmentally sound approach to reduce stripe rust damage. To better understand the APR to wheat stripe rust, we investigated the change of photosynthetic characteristics and antioxidant systems in susceptible and resistant wheat cultivars that were infected by wheat stripe rust.

It is well known that different stressful environments usually cause substantial damage to photosynthetic pigments (Ashraf and Harris, 2013). The decrease in total Chl content is a common phenomenon under biotic and abiotic stresses. The stress-induced alterations in leaf Chl content may be due to impaired biosynthesis or accelerated pigment degradation (Perveen et al., 2010). In consistence with these previous studies, we found that the stripe rust infection resulted in a decrease in the Chl content in both the susceptible and the resistant wheat, especially the susceptible wheat. This result indicates that the resistant wheat has a more effective protective system against damages to pigments caused by the infection. In addition, the photosynthetic rates were significantly decreased at 72 hpi, likely because pathogen infection can reduce green leaf areas or damage mesophyll cells (Chang et al., 2013).

ROS species are generated in cells during plant–pathogen interactions and are associated with resistance to stripe rust, as reported in previous studies using histological or microarray methods (Wang et al., 2007; Coram et al., 2008). The results from the present study showed that stripe rust markedly induced ROS accumulation in the leaves of inoculated wheat plants, particularly in the susceptible wheat (**Figure 3**). This finding indicates a relationship between increased cell death and ROS accumulation. It has been shown that ROS plays a dual role in plant–pathogen interaction (Feng et al., 2014), however the role of ROS in plant defense responses depends on its concentration (Mittler et al., 2004). A low ROS concentration induces protective antioxidant mechanisms and triggers a systemic response, while a moderate or high ROS concentration can be toxic to pathogens (Peng and Kuc, 1992; Solomon et al., 1999). Here, we confirmed that the protective antioxidant system was activated in the resistant wheat, which subsequently changed the levels of ROS and avoided severe oxidative damages caused by ROS overproduction. The accumulation of $O_{2}^{\cdot –}$ and H_2_O_2_ is usually observed in the incompatible interaction during the early stage of pathogen infection (Wang et al., 2007). However, the study of Wang et al. (2007) also showed that H_2_O_2_ accumulation was observed in mesophyll cells in the compatible interaction at the late stage of 96 h after inoculation, which is consistent with our results of 72 h after inoculation during the boot stage. Therefore, the ROS accumulation level likely also depends on the inoculated time and varies in different wheat cultivars. During environment stresses, ROS species are also reportedly involved in lipid peroxidation, which in turn result in membrane injury (Smirnoff, 1993). The high content of $O_{2}^{\cdot –}$ and H_2_O_2_ in the susceptible wheat after infection could be estimated by the accumulation of MDA and the electrolyte leakage (Halliwell and Gutteridge, 1984). In this study, our results indicated that ROS may play an important role in plant resistance to stripe rust during the adult plant stage.

To detoxify the excessive ROS species accumulation, plants have evolved a complex antioxidant defense system to eliminate or reduce their damaging effects. Previous studies indicated that stripe rust infection leads to the activation of some antioxidant enzymes in resistant wheat genotypes; by contrast, these enzymes are inhibited in the susceptible genotypes (Ivanov et al., 2005; Asthir et al., 2010). The activity of several antioxidant enzymes was markedly induced in susceptible barley after powdery mildew inoculation, while less pronounced pathogen-induced increases were detected in inoculated leaves of resistant plants (Harrach et al., 2008). In the present study, pronounced differences in these antioxidant enzyme activities were observed in susceptible and resistant wheat cultivars. After infection, the activity of POD, CAT, and GPX was markedly increased in resistant wheat compared with susceptible wheat, suggesting that these three antioxidant enzymes in inoculated adult plants might play an important role in regulating the levels of ROS. In contrast, pronounced decreases in the activity of SOD, APX, and GR were found in susceptible wheat after the stripe rust infection. This may be due to the severe damages in the susceptible wheat.

The common approach for screening genotypes for disease resistance is costly and time-consuming, and also depends on the environmental conditions. Therefore, the development of rapid, accurate and objective evaluation methods is urgently needed in agriculture. Chlorophyll fluorescence has been proven to be a useful, non-invasive tool for the study of different aspects of photosynthesis, as well as for the detection of various environmental stresses in a wide range of plant species (Ashraf and Harris, 2013). A previous study indicated that measurement to the quantum yield of non-regulated energy dissipation in PSII is a valuable tool for screening wheat plants for leaf rust resistance (Burling et al., 2010). In the present study, the susceptible cultivar showed a stronger decline of chlorophyll fluorescence compared with the resistant cultivar. In the susceptible wheat, the lower *F*_v_/*F*_m_ value may be due to partial inactivation of the PSII reaction centers. At the same time, the lower qP and ΦPSII also reflect a lower quantum yield of PSII in the susceptible wheat cultivar. In addition, the higher NPQ in the susceptible wheat shows the increased need of dissipating excess light energy, possibly because of its lower PSII activity. Furthermore, observations of the thylakoid membrane ultrastructure also indicated that the stripe rust infection alters grana stacking and relaxation of the thylakoid structure (**Figure 9**), thereby resulting in a decrease in PSII activity in the susceptible wheat plants. Hence, the results suggest that the resistant cultivar avoids PSII damages more effectively than the susceptible cultivar.

To date, most studies have been conducted on pathogen-resistant (PR) genes or proteins. There are few studies on effects of the stripe rust infection on photosynthetic proteins. A previous study showed that stripe rust infection may result in a decrease in D1 protein in susceptible wheat (Shen et al., 2008). This finding is consistent with our results, in which the content of D1 was reduced in the susceptible wheat leaves, but increased in the resistant wheat plants when challenged with *Pst*. The levels of PSI and other PSII proteins were not changed in both the susceptible and the resistant wheat plants, indicating that most photosynthetic proteins do not participate in the regulation of plant resistance to stripe rust during the adult plant stage. In addition, our study indicated that CP29 was strongly phosphorylated in the resistant wheat plants after the stripe rust infection, suggesting that CP29 phosphorylation plays an important role in plant resistance. Previous studies showed that CP29 protein phosphorylation in thylakoid membranes may be involved in a number of responses to a changing environment (Chen et al., 2009; Liu et al., 2009).

Although there are no studies on thylakoid protein phosphorylation after stripe rust infections, phosphorylation and dephosphorylation of PSII proteins have been reported to play an important role in the response to environmental stresses (Aro and Ohad, 2003; Vener, 2007; Liu et al., 2009; Fristedt et al., 2010). In the current study, we found that D1 and LHCII proteins were strongly phosphorylated in the resistant wheat plants after the stripe rust infection, suggesting that phosphorylation of D1 and LHCII may be also involved in plant defense.

## Summary

We show that stripe rust infections markedly altered the photosynthetic characteristics and antioxidant systems in susceptible and resistant wheat cultivars. We found that the resistant wheat may be effective in alleviating excessive ROS through antioxidant enzymes and may maintain a higher PSII activity compared with the susceptible wheat plants. In addition, strong PSII protein phosphorylation was also observed in the resistant wheat. Based on these results, we propose that the antioxidant enzymatic systems and PSII protein phosphorylation may play an important role in plant resistance to stripe rust.

## Conflict of Interest Statement

The authors declare that the research was conducted in the absence of any commercial or financial relationships that could be construed as a potential conflict of interest.
